# Supplementary material for: Mining Candidate Genes Related to Heavy Metals in Mature Melon (Cucumis melo L.) Peel and Pulp Using WGCNA
Source: Genes (Basel). 2022 Sep 30;13(10):1767. doi: 10.3390/genes13101767 (PMC9602089; doi:10.3390/genes13101767)
Supplement: Supplementary file 1 [file genes-13-01767-s001.zip › genes-1883479-supplementary/Supplementary materials.pdf]

Supplementary materials:

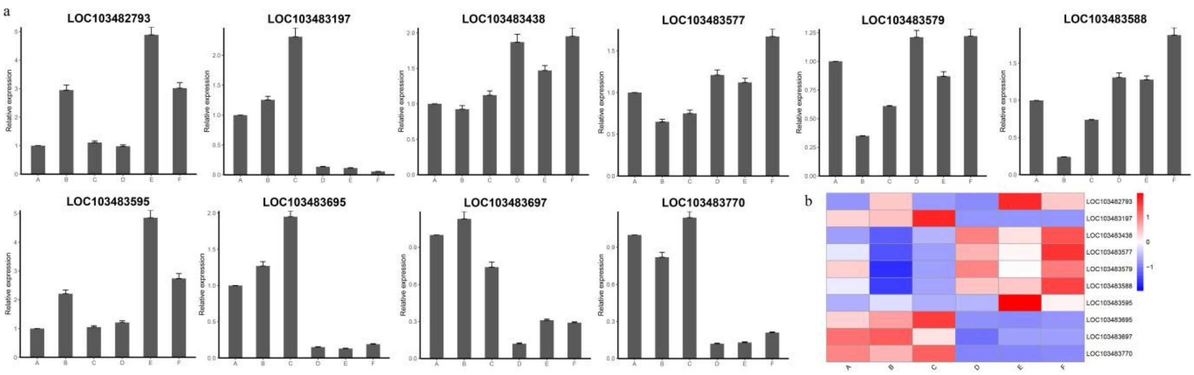

Figure S1: (a) Analysis of 10 genes in 6 tissues by qRT-PCR; (2) Analysis of 10 genes expression in 6 tissues.

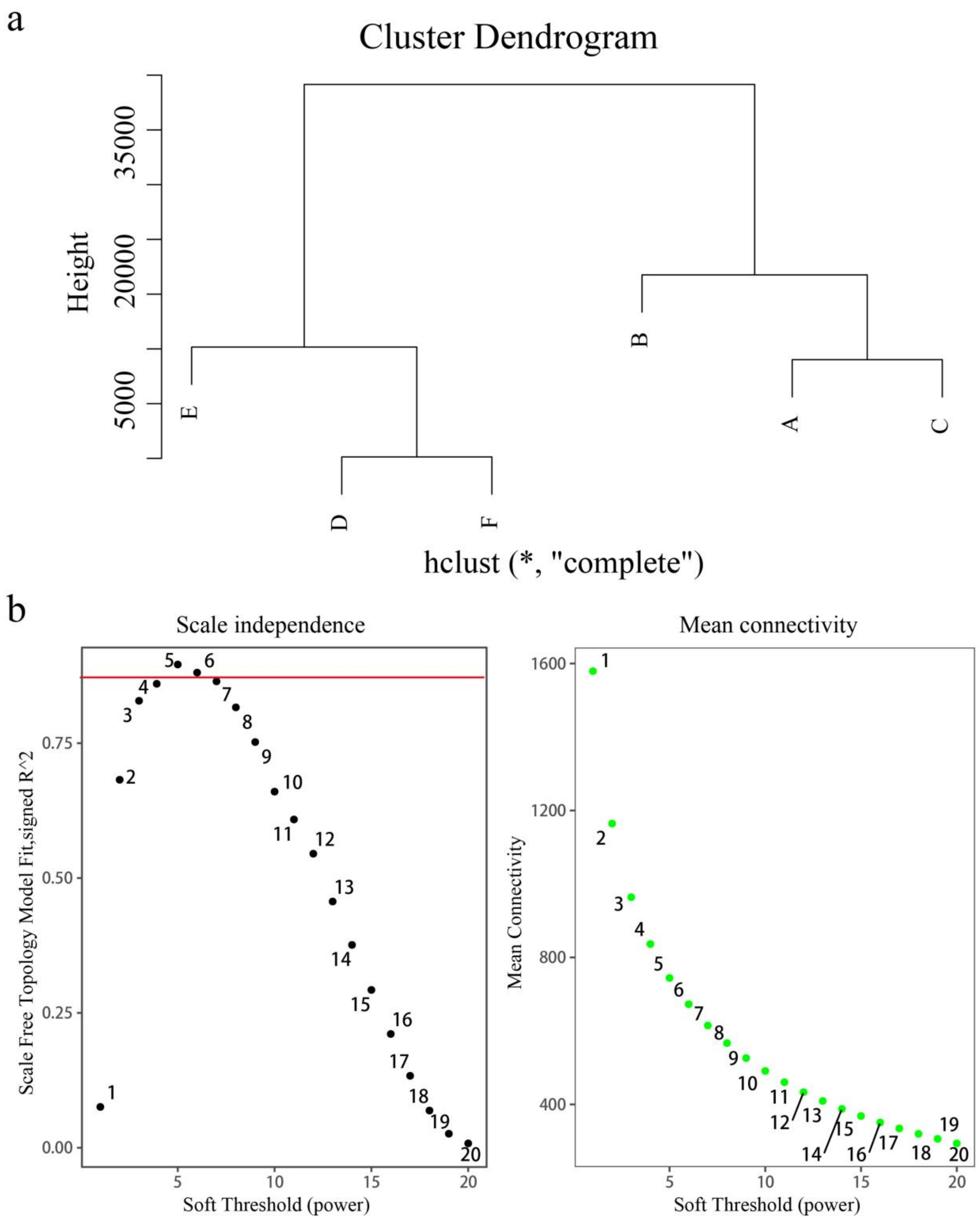

Figure S2: (a) WGCNA sample hierarchical clustering; (2) The power value of WGCNA to build a scale-free network.

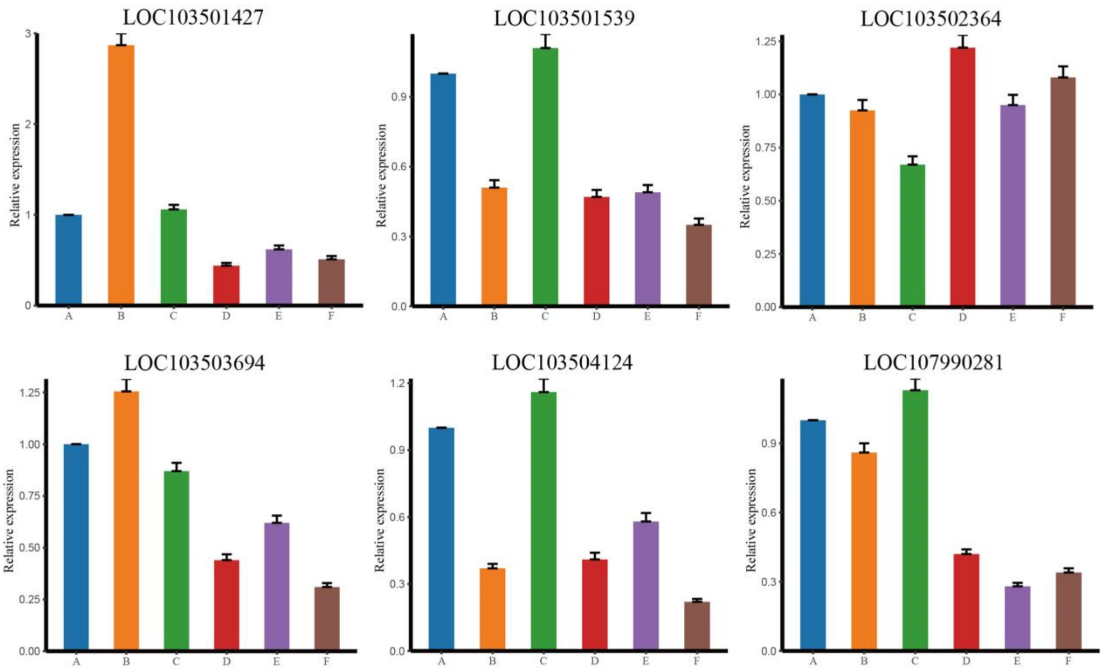

Figure S3: qRT-PCR analysis of six hub genes in six tissues.

Table S1: All primers used in this study.

gene_id	Forward primer	Reverse primer
LOC103482793	GAGCTTTCCATTAGCCCACTA	TTAGGGATTGAAACGGCAGAG
LOC103483197	TATGAGTCATATTTGTACT	GAAATCTTAAATCAATAAC
LOC103483438	CATAACACTGCCTCATTGACA	AACAGATTATGAGCTGCTACA
LOC103483577	CTCGTGCCTGTACAGAAGCC	ACGATCAGGAGCAAGCTTCA
LOC103483579	AGCCTCAAGGACCTCTCGTT	TCCCAATCTGCCAGTCCTTTC
LOC103483588	GATCCGGCGCTGAGATTACA	AAGATGCGGTGATGAAGCCA
LOC103483595	AGCTGAGGCGGCTAGGATTA	TCCGAAATCCGCCAGTTTCA
LOC103483695	GTTGCGGTTATCGTTGCTGT	GCTGTGTGCAGCATGATTGT
LOC103483697	ACAACGTCGATTCACAGCCA	AGTATCGAGCGGCTTTCTCG
LOC103483770	TGGCAGTTACGGTCGAACAA	CATGGAGTGGAGGGTCCAAC
LOC103501427	CTCATATCCGCTTCCGCCAT	CAGTGATCTCCACGCCACAT
LOC103501539	CTTGCAATGGCTGAAACGCT	ACTGAATCACCAGAGCCGTG
LOC103502364	AGTGAATTCCCTCCACCGTTC	AAGGAAGAGGCGGCTTATGG
LOC103503694	TCCATGCCGGAAAACCCATC	CTTCTTCCCCTTCCGCGATA
LOC103504124	TGGATTTGGCGTACGGGTTC	AAGGACTTTCACGTCCACCA
LOC107990281	ATTTGGCGTACGGGTTCCTT	AGCCGGGGGATTGTATTTGG
CmActin	CAAGGGCCGTCTTCCCTAGC	GCTTTGGGATTGAGTGGTGC

Table S2: Samples and libraries used for the de novo and reference-based transcriptomic analyses.

Sample	Raw reads (million)	Clean reads (million)	total bases(Gb)	Q20 percentage (%)	Q30 percentage (%)	GC content(%)	alignment rate(%)
A-1	71.87	70.13	7.34	93.99	87.41	44.44	96.53
A-2	71.87	69.88	7.02	94.06	87.48	45.18	96.18
A-3	71.87	70.06	7.33	94.01	87.39	45.08	94.22
B-1	73.62	71.06	7.44	94.22	87.82	45.93	96.05
B-2	71.87	69.87	6.96	93.96	87.23	44.77	97.13
B-3	71.87	69.94	6.87	93.92	87.18	44.47	97.28
C-1	73.62	70.93	7.07	93.97	87.34	44.54	94.55
C-2	71.87	70.11	7.14	94.01	87.36	44.32	96.23
C-3	71.87	69.91	6.99	93.89	87.19	44.76	95.18
D-1	73.62	70.82	6.93	93.97	87.34	45.23	94.77
D-2	71.87	69.57	6.87	94.22	87.81	44.79	96.58
D-3	71.87	70.09	7.12	93.98	87.31	44.88	95.28
E-1	71.87	70.05	7.05	94.16	87.72	44.93	93.18
E-2	71.87	69.74	6.99	94.01	87.37	45.08	94.25
E-3	71.87	69.82	7.01	93.95	87.32	45.12	94.19
F-1	71.87	70.58	7.03	93.71	86.74	44.97	95.44
F-2	71.87	69.81	6.88	94.02	87.38	44.88	95.87
F-3	71.87	69.92	7.05	93.98	87.31	45.01	96.22
