# Supplementary figures and images for: Mining Candidate Genes Related to Heavy Metals in Mature Melon (Cucumis melo L.) Peel and Pulp Using WGCNA
Source: Genes (Basel). 2022 Sep 30;13(10):1767. doi: 10.3390/genes13101767 (PMC9602089; doi:10.3390/genes13101767)

a

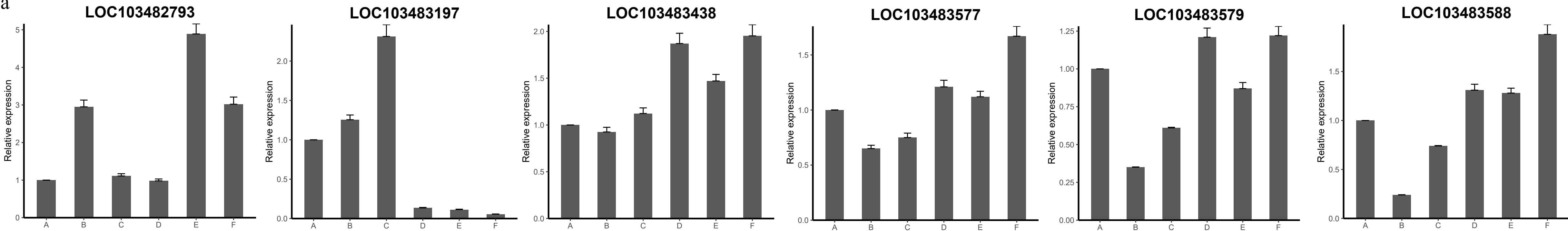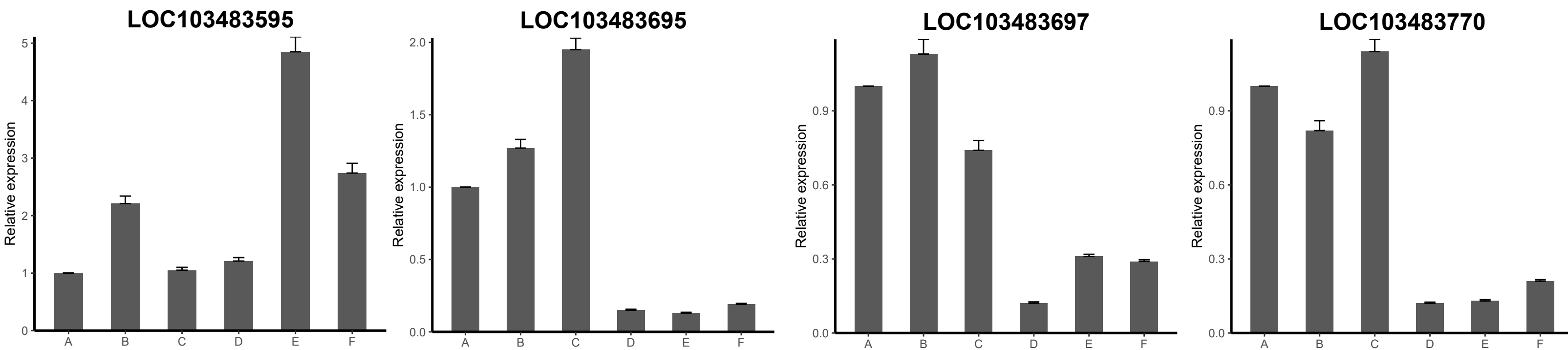

b

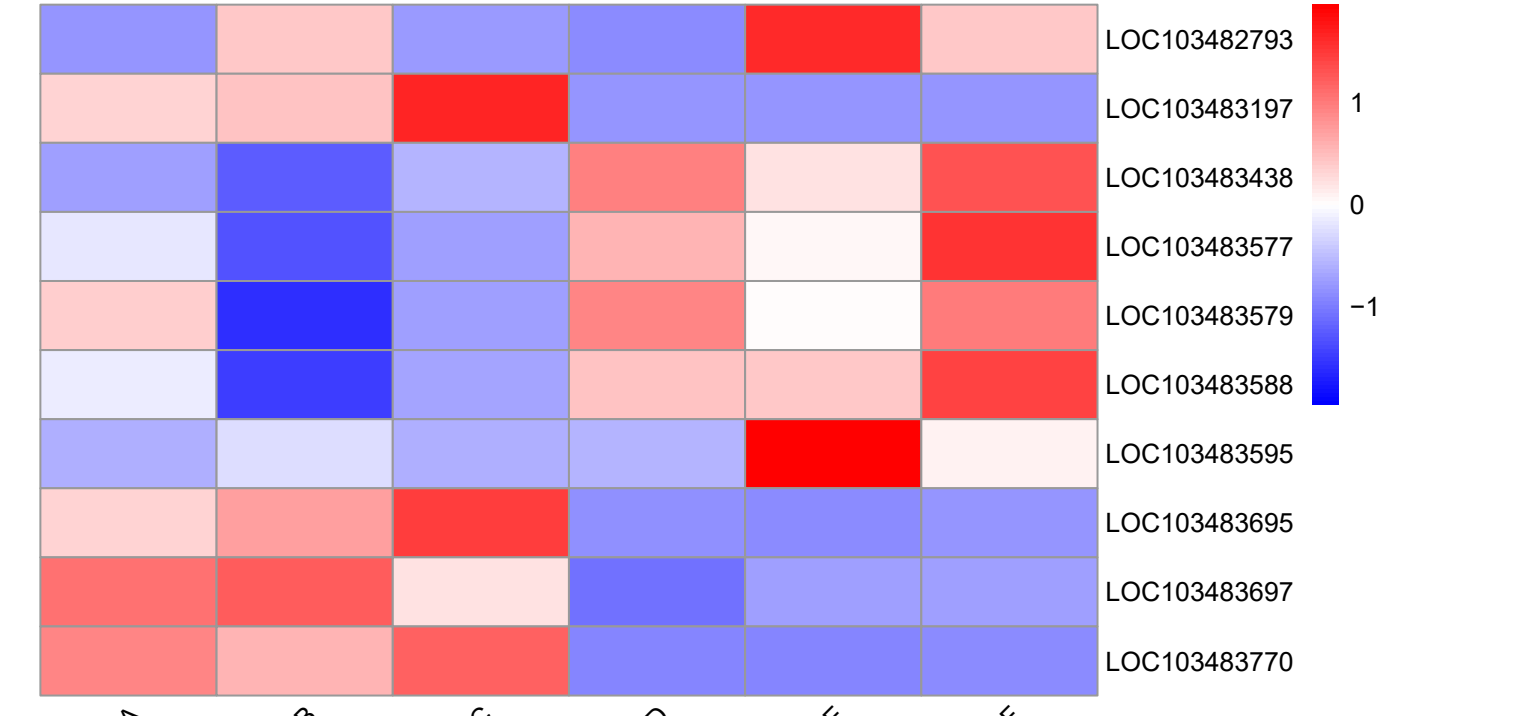

Supplement: Supplementary file 1 [file genes-13-01767-s001.zip › genes-1883479-supplementary/Fig S1.pdf]

a

## Cluster Dendrogram

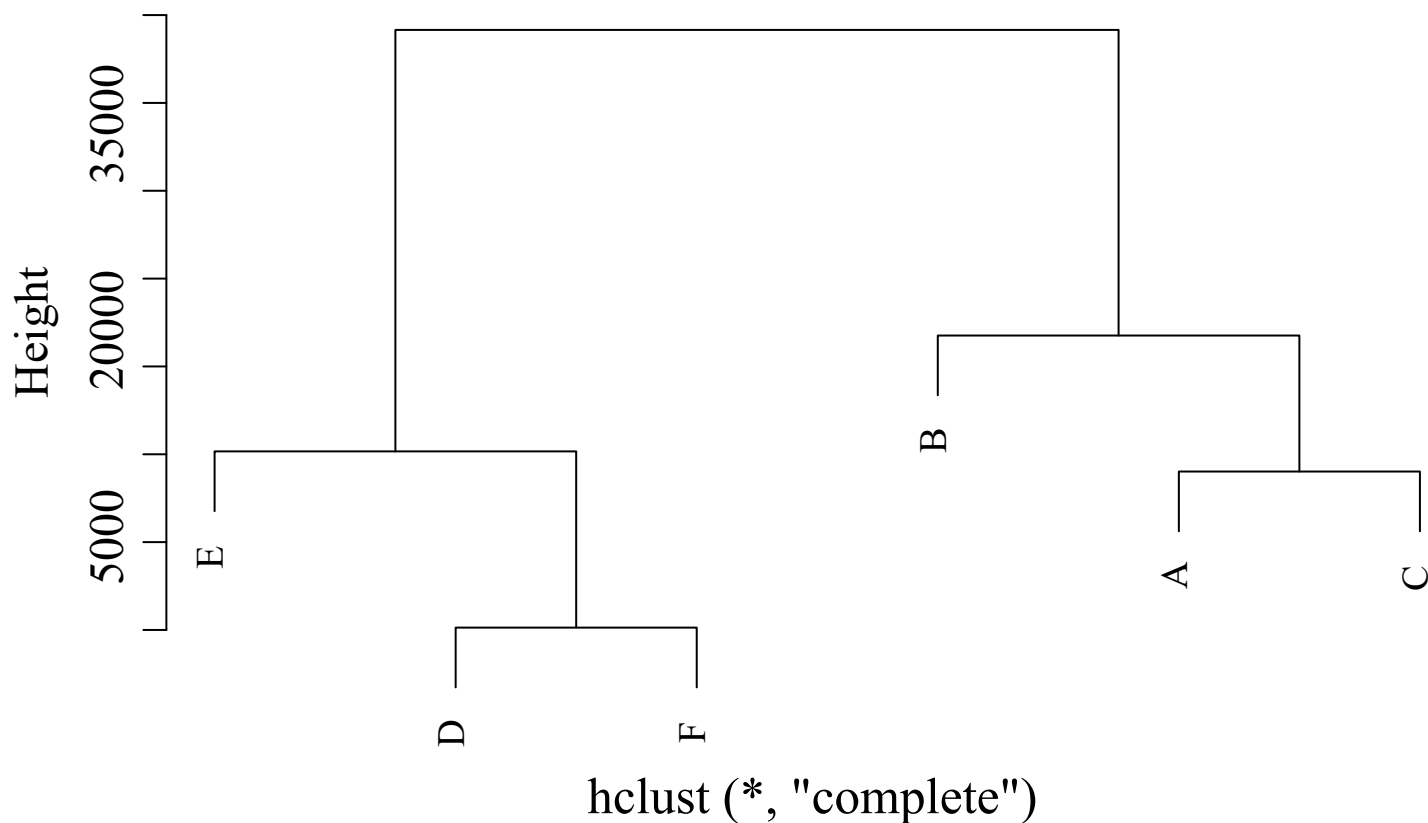

b

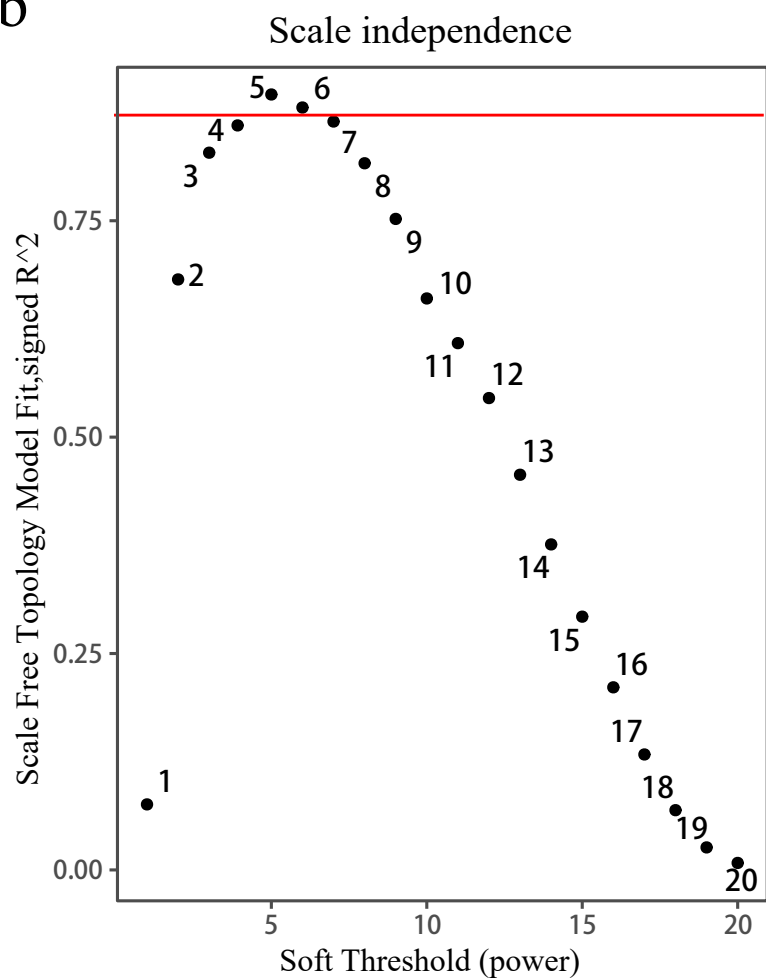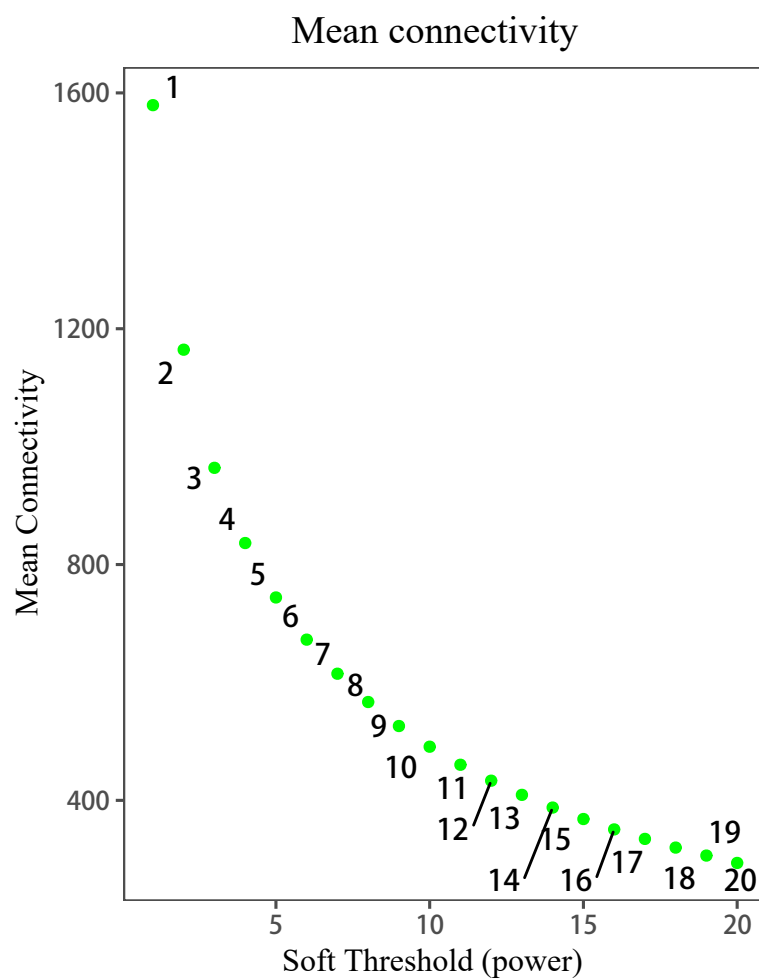

Supplement: Supplementary file 1 [file genes-13-01767-s001.zip › genes-1883479-supplementary/Fig S2.pdf]

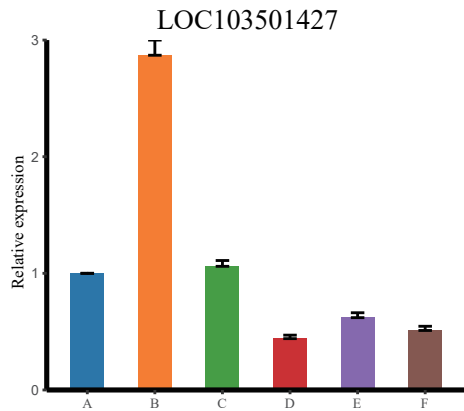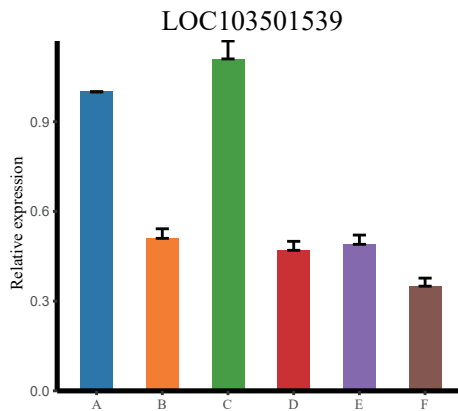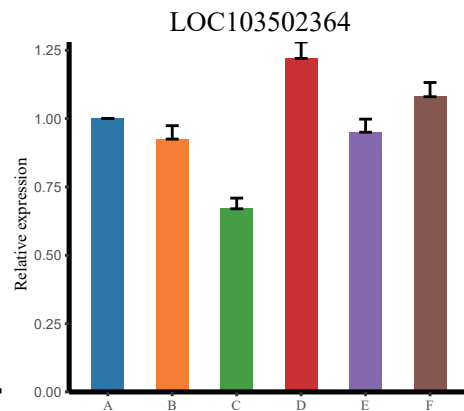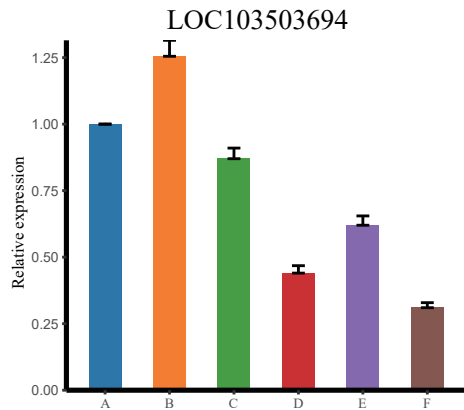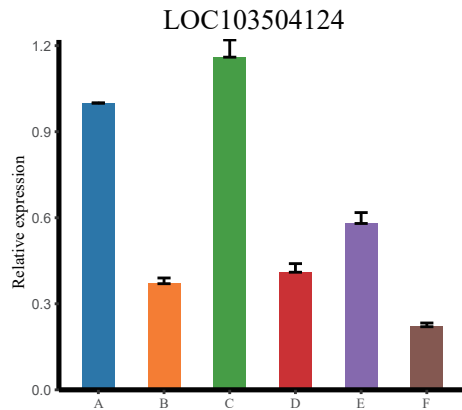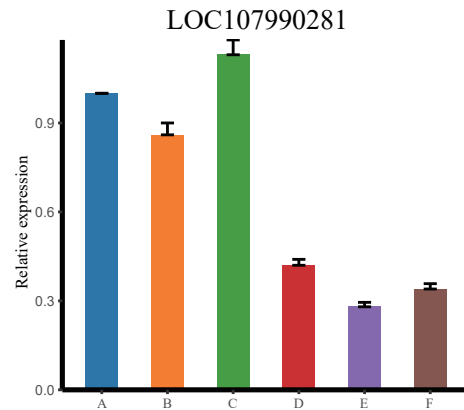

Supplement: Supplementary file 1 [file genes-13-01767-s001.zip › genes-1883479-supplementary/Fig S3.pdf]
